# *Bifidobacterium animalis* subsp. *lactis* Ca360 Promotes Oral Iron Repletion, Alters the Gut Microbiota, and Regulates Host Metabolism and Inflammatory Status in a Murine Model of Iron Deficiency Anemia Caused by a Low-Iron Diet

**DOI:** 10.3390/nu18060900

**Published:** 2026-03-12

**Authors:** Peiqing Jiang, Jing Yang, Yuejian Mao, Linjun Wu, Xiaoqiong Li, Xiangyu Bian, Jian Kuang, Jianqiang Li, Fangshu Shi, Xiaoqiang Han, Jinjun Li, Haibiao Sun

**Affiliations:** 1 First Clinical Medical College, Shanxi Medical University, Taiyuan 030000, China; jiangpeiqing577@163.com (P.J.); jack98193@163.com (X.H.); 2 State Key Laboratory for Quality and Safety of Agro-Products, Institute of Food Sciences, Zhejiang Academy of Agricultural Sciences, Hangzhou 310021, China; 2112005190@zjut.edu.cn (L.W.); lixiaoqiong@zaas.ac.cn (X.L.); bianjinfeng1993@163.com (X.B.); jian_kuang95@yeah.net (J.K.); jianqiang.li910@outlook.com (J.L.); shifangshu1992@163.com (F.S.); 3 Global R&D Innovation Center, Inner Mongolia Mengniu Dairy (Group) Co., Ltd., Hohhot 011500, China; yangjing1119@mengniu.cn (J.Y.); maoyuejian@mengniu.cn (Y.M.)

**Keywords:** IDA, iron absorption, iron metabolism, *B. lactis* Ca360, gut microbiota

## Abstract

**Background/Objectives:** Iron deficiency anemia (IDA) is a widespread nutritional disorder characterized by impaired iron absorption, inflammation-associated iron restriction, and disrupted iron homeostasis. Increasing evidence suggests that gut microbiota play an important role in iron metabolism; however, the mechanisms underlying probiotic-assisted iron supplementation remain unclear. Our research group previously conducted in vitro fermentation screening experiments and obtained a bacterial strain, *B. lactis* Ca360, which possesses iron absorption-enhancing activity. **Methods:** In this study, an IDA mouse model induced by a low-iron diet was used to investigate whether *B. lactis* Ca360 could synergistically improve iron metabolism when combined with iron supplementation. Mice were treated with FeSO_4_ alone or FeSO_4_ combined with *B. lactis* Ca360, and hematological parameters, organ indices, serum iron-related markers, histopathological changes, duodenal iron metabolism-related gene expression, hepatic inflammatory responses, gut microbiota composition, short-chain fatty acid (SCFA) levels, and correlation networks were analyzed. **Results:** Iron deficiency induced typical anemia phenotypes, multi-organ dysfunction, intestinal iron absorption dysregulation, hepatic inflammation, and gut microbiota dysbiosis. Compared with FeSO_4_ alone, the combined intervention more effectively improved hematological parameters, reduced organ indices, restored liver and spleen histological integrity, normalized intestinal iron metabolism-related gene expression, and alleviated hepatic inflammation. In addition, *B. lactis* Ca360 markedly reshaped gut microbiota composition, enriching SCFA-producing anaerobic genera, including *Ruminococcus*, *Roseburia*, *Acetatifactor*, *Intestinimonas*, *Eubacterium_coprostanoligenes_group_unclassified*, and *Oscillibacter*, accompanied by increased acetate, propionate, and butyrate levels. Spearman correlation analysis further revealed close associations between gut microbiota remodeling, improved iron metabolism, reduced inflammatory status, and recovery of anemia-related phenotypes. **Conclusions:** Overall, these findings demonstrate that *B. lactis* Ca360 enhances the efficacy of iron supplementation by modulating SCFA-producing and anti-inflammatory gut microbiota, thereby coordinately regulating intestinal iron absorption, inflammation, and systemic iron homeostasis, supporting probiotic-assisted iron supplementation as a promising nutritional strategy for IDA management.

## 1. Introduction

Iron is an essential trace element for living organisms, participating in numerous critical biological processes including oxygen transport, DNA synthesis, and cellular energy metabolism [1]. However, iron deficiency and the resulting iron deficiency anemia (IDA) remain the most prevalent nutritional disorders globally. According to the World Health Organization, approximately 42% of children and 40% of pregnant women worldwide suffer from anemia, half of which can be attributed to iron deficiency [2]. This not only leads to cognitive impairment and stunted growth but also imposes a heavy socioeconomic burden [3]. Currently, oral inorganic iron supplements (e.g., ferrous sulfate, FeSO_4_) serve as the primary clinical treatment for IDA. However, conventional iron supplementation strategies present substantial limitations. In the alkaline intestinal environment, iron supplements readily form insoluble precipitates, leading to poor absorption efficiency and low bioavailability [4]. In addition, oral iron intake is frequently associated with gastrointestinal adverse effects, including nausea, constipation, and abdominal pain, which markedly compromise patient compliance [5]. Unabsorbed iron ions can also disrupt intestinal microecological homeostasis by promoting the proliferation of pathogenic bacteria, such as members of the family *Enterobacteriaceae* [6], while suppressing beneficial genera such as *Lactobacillus* and *Bifidobacterium*, thereby exacerbating gut dysbiosis [7]. Moreover, non-physiological iron absorption may disturb systemic iron homeostasis, increasing the risk of iron overload and triggering oxidative stress and organ damage [8]. Collectively, these limitations underscore the urgent need for the development of safer and more effective iron supplementation strategies.

In recent years, the role of the gut microbiota in regulating systemic iron homeostasis has gained increasing prominence. Studies indicate that gut bacteria ferment dietary fiber to produce SCFAs, which lower colonic pH and enhance iron solubility and absorption [9]. Concurrently, specific probiotics can directly express iron-binding proteins to facilitate iron transport across cell membranes [10]. Conversely, iron deficiency can also lead to dysbiosis. It alters the intestinal iron microenvironment by reducing luminal iron availability and enhancing host iron-sequestration responses, thereby intensifying microbial competition for this essential micronutrient and favoring microorganisms equipped with high-affinity iron acquisition systems, such as siderophore- or heme-mediated uptake pathways and iron-responsive regulatory mechanisms [11]. These selective pressures may lead to reduced microbial diversity and shifts in community composition, frequently characterized by a relative depletion of beneficial commensal bacteria, including *Lactobacillus* and *Bifidobacterium* or other short-chain fatty acid-producing taxa, accompanied in certain contexts by an increased abundance of opportunistic or pathobiont species [12]. Such alterations in microbial community structure may impair the production of metabolites important for intestinal homeostasis, including short-chain fatty acids, and potentially weaken barrier-supportive functions, thereby exacerbating dysbiosis and contributing to the vicious cycle of “iron deficiency–microbiome disruption–impaired iron absorption” [13]. Therefore, targeting the regulation of the gut microbiota is considered a potential new approach to break this cycle and improve iron absorption. Among these, probiotics have garnered significant attention due to their high safety profile and ease of access and application. Potential mechanisms by which probiotics may improve iron metabolism include competitive inhibition of pathogenic bacteria, thereby reducing microbial iron consumption [14], as well as the production of organic acids that enhance iron solubility and bioavailability [15]. In addition, probiotics may help maintain intestinal barrier integrity [16], limiting iron loss [17], and optimize systemic iron distribution by modulating the expression of hepcidin—the key iron-regulatory hormone produced mainly by hepatocytes—which binds to the iron exporter ferroportin and induces its internalization and degradation, thereby inhibiting cellular iron release into the circulation [18]. *Lactobacillus plantarum* 299v (*L. plantarum* 299v), owing to its unique role in promoting iron absorption, has been widely used as a model strain in iron metabolism research. A randomized controlled trial showed that its supplementation significantly increased non-heme iron absorption, with the effect positively correlating with dosage [19]. Several mechanisms have been proposed to explain how *L. plantarum* 299v facilitates host iron metabolism. First, this strain produces lactic acid and other organic acids that lower intestinal luminal pH, thereby increasing the solubility and bioavailability of non-heme iron [20]. Second, *L. plantarum* 299v may enhance host iron uptake by modulating intestinal iron transport pathways. In vitro studies further revealed that this strain upregulated the expression of *DcytB*, a ferric reductase encoded by the *Cybrd1* gene, in intestinal epithelial cells [21]. *DcytB* is localized on the brush-border membrane of enterocytes and functions as a key membrane-bound ferric reductase that reduces luminal non-heme iron from Fe^3+^ to Fe^2+^ [22]. Fe^2+^ serves as the preferred substrate for *DMT1*-mediated transmembrane transport, thereby facilitating intestinal iron uptake. Additionally, it efficiently colonizes the intestinal mucosa, outcompeting iron-sequestering pathogens. This reduces microbial iron consumption, indirectly increasing iron availability for the host [23]. Although some studies have shown that certain probiotics can promote iron absorption, the number of such strains remains limited. Existing research primarily focuses on the relationship between microbial community changes and iron absorption efficiency, lacking comprehensive evaluation of systemic effects such as regulation of overall iron homeostasis, improvement of hematological indicators, and organ repair. Furthermore, the overall advantages of probiotics compared to traditional iron supplementation need further clarification. Therefore, screening and validating novel probiotics and their mechanisms of action holds significant research value.

Building upon our prior successful screening of *Bifidobacterium animalis* subsp. *lactis* Ca360 (*B. lactis* Ca360) with high iron adsorption capacity, this study aims to systematically investigate its efficacy and potential mechanisms in treating IDA. We hypothesize that *B. lactis* Ca360 may achieve safe and effective correction of anemia by reshaping the gut microbiota to synergistically enhance iron absorption, recycling, and storage. This study systematically evaluated the therapeutic efficacy and safety advantages of *B. lactis* Ca360 over FeSO_4_ by integrating multidimensional evidence from microbiomics, hematology, iron metabolism indicators, and histopathology. It not only provides a robust theoretical basis for probiotic applications in treating nutritional anemia but also reveals for the first time a potential mechanism whereby *B. lactis* Ca360 functions through the “gut–blood–organ” regulatory axis.

## 2. Materials and Methods

### 2.1. Materials and Reagents

Anticoagulant blood tubes (EDTA-K2) were purchased from Labshark (Changde, China). Phosphate-buffered saline (PBS) was bought from Solarbio (Beijing, China). Paraformaldehyde tissue fixation solution (4%) was provided by Servicebio (Wuhan, China). Ferrous sulfate was purchased from Shanghai Lingfeng Chemical Reagent Co., Ltd. (Shanghai, China). The normal standard diet (catalogue No.: XTI01WC-004, containing 100 mg Fe/kg) and iron-deficient diet (catalogue No.: XT19008, containing 4 mg Fe/kg) were purchased from Xietong Bioengineering Co., Ltd. (Nanjing, China). Both diets were isocaloric (3886 kcal/kg) and matched for macronutrients (protein: 20.6%, fat: 12.0%, carbohydrate: 67.4%). The normal standard diet was an irradiation-sterilized maintenance diet specifically formulated for experimental mice, with a consistent composition and complete nutrition including adequate iron content, and was in compliance with national standards GB/T 14924.1, GB/T 14924.2, GB 14924.3, and GB 13078 [24,25,26,27]. The detailed ingredient compositions of both diets are available on the official website of Xietong Bioengineering Co., Ltd. The iron-deficient diet was a customized purified irradiation-sterilized diet with low iron content, which was insufficient to meet the normal physiological iron requirements of mice.

### 2.2. Preparation of B. lactis Ca360 Suspension

*B. lactis* Ca360 was supplied by Inner Mongolia Mengniu Dairy (Group) Co., Ltd. (Hohhot, China). The strains were isolated from a pediatric intestinal sample and stored in cryovials containing 30% (*v*/*v*) glycerol, kept at −80 °C. Prior to experiments, the strain was inoculated into MRS broth medium and activated for 24 h in an anaerobic incubator at 37 °C. The bacterial cells were collected by centrifugation at 8000 rpm for 5 min at 4 °C. After discarding the supernatant, the bacterial pellet was washed twice with pre-chilled phosphate-buffered saline (PBS, pH 7.4) and finally resuspended in the same buffer. To ensure the final concentration of the bacterial suspension accurately reached 1 × 10^9^ CFU/mL [28], the McFarland densitometer (Grant-bio, Cambridge, UK) was used for turbidity determination, and the dilution was performed according to the formula C_1_V_1_ = C_2_V_2_ (where C_1_ represents the initial concentration of the bacterial suspension, V_1_ represents the volume of the initial bacterial suspension, C_2_ represents the target final concentration of 1 × 10^9^ CFU/mL, and V_2_ represents the final volume of the bacterial suspension after dilution) [29]. To validate the accuracy of the McFarlan-based concentration adjustment, the prepared suspension was serially diluted and plated onto MRS agar. Following anaerobic incubation at 37 °C for 48 h, colonies were enumerated to confirm that the actual concentration of the bacterial suspension was consistent with the target 1 × 10^9^ CFU/mL. The bacterial suspensions were prepared fresh daily for animal gavage.

### 2.3. Experimental Animal Model of Iron Deficiency Anemia

The animal study was reviewed and approved by the Ethics Committee of the Zhejiang Academy of Agricultural Sciences (approval NO: 25ZALAS11). Fifty healthy male-specific pathogen-free (SPF) Institute of Cancer Research (ICR) strain mice aged three weeks were provided by Jiangsu Jicui Yao Kang Biotechnology Co., Ltd. (Nanjing, China). All mice were housed in a temperature-controlled room (22 ± 2 °C and 50 ± 10% relative humidity) with a 12 h light/dark cycle. During the experiments, all mice were allowed access to food and sterile water ad libitum. All animal experiments were conducted under SPF conditions to minimize environmental contamination.

After a 7-day acclimatization period, the mice were randomly divided into five groups (*n* = 10 per group): (1) normal control group (NC, standard diet); (2) iron deficiency anemia model group (IDA, iron-deficient diet); (3) FeSO_4_ group (iron-deficient diet + FeSO_4_ at 3 mg iron/kg BW/day) [30]; (4) FeSO_4_ + *L. plantarum* 299v group (iron-deficient diet + *L. plantarum* 299v at 1 × 10^9^ CFU/mL + FeSO_4_ at 3 mg iron/kg BW/day), and (5) FeSO_4_ + *B. lactis* Ca360 group (iron-deficient diet + *B. lactis* Ca360 at 1 × 10^9^ CFU/mL + FeSO_4_ at 3 mg iron/kg BW/day). Body weight was recorded weekly. Based on the basic iron requirement of 3 mg iron/kg body weight (BW), FeSO_4_·7H_2_O powder was weighed and dissolved in sterile water to prepare FeSO_4_ solution for mouse gavage administration. To ensure the stability and efficacy of the solution, the FeSO_4_ solution was prepared freshly each day immediately prior to gavage, thereby preventing oxidation and maintaining transparency throughout the administration period. Bacterial suspensions and FeSO_4_ solution were freshly prepared in sterile water for oral gavage, and the administration volume was 0.2 mL per mouse for all treatments. Mice in both NC and IDA groups received an equivalent volume of sterile water. Blood samples were collected weekly from the tail vein into EDTA anticoagulant tubes. Mice were considered a model of iron deficiency anemia (IDA) when hemoglobin levels of the model group decreased below 100 g/L [31]. The animals were gavaged once daily between 10:00 and 12:00 a.m. for 7 weeks.

### 2.4. Organ Coefficient

After dissecting the mice, the heart, liver, spleen, and kidney were carefully separated, blotted dry with absorbent filter paper to remove residual blood and interstitial fluid (to avoid interference with weight measurement), and then accurately weighed using an electronic balance with a precision of 0.1 mg. Multiple measures were taken to reduce variation throughout the procedure: first, all dissection operations were performed by the same trained operator to ensure consistency in organ separation and blotting methods; second, the electronic balance was calibrated with standard weights before each measurement session to ensure measurement accuracy; third, each organ was weighed twice consecutively, and the average value of the two measurements was used as the final organ weight (if the relative deviation between the two measurements exceeded 0.5%, the weighing was repeated until the deviation met the requirement); and fourth, all filter papers used for blotting were of the same brand and specification to avoid differences in water absorption capacity affecting the blotting effect. The relative weight of each organ was calculated from the final body weight of each individual mouse measured on the day of dissection (rather than the average body weight of the group). The organ coefficients were calculated as follows [32]:

$$Organ\;idex=\frac{Organ\;weight\;(g)}{Body\;weight\;(g)}\times 100\%$$

The final body weight of each mouse was used for normalization to more accurately reflect the relative weight of each organ in the individual animal, which helps to reduce the interference of individual differences in body weight within the group on the organ coefficient calculation.

### 2.5. Hematological Test

Blood samples were collected into EDTA-coated anticoagulant tubes and analyzed immediately (within 30 min after collection) using an automated hematology analyzer (BC-5000 Vet, Mindrayanimal, Shenzhen, China) for routine hematological indices, including hemoglobin (HGB), red blood cell count (RBC), mean corpuscular volume (MCV), and hematocrit (HCT). Each sample was analyzed in triplicate technical replicates, with *n* = 10 biological replicates per group. Quality control was performed once weekly in accordance with the manufacturer’s instructions and standard laboratory protocols.

### 2.6. Enzyme-Linked Immunosorbent Assay (ELISA)

Serum levels of erythropoietin (EPO), ferritin, hepcidin, and soluble transferrin receptor (sTfR) were determined using commercial ELISA kits purchased from Jiangsu Jingmei Biological Technology Co., Ltd. (Yancheng, China), with the following catalog numbers: EPO (JM-02477M1), ferritin (JM-02595M1), hepcidin (JM-12030M1), and sTfR (JM-13460M1). The detection ranges for each kit were as follows: EPO, 0.75–24 IU/L; hepcidin, 0.5–16 ng/mL; ferritin, 5–160 ng/mL; and sTfR, 2.5–80 nmol/L. The sensitivity of all ELISA kits was defined as a minimum detectable concentration of <0.1 unit, except for ferritin, which had a minimum detectable concentration of <1 unit. All kits showed high specificity, with no cross-reactivity with other soluble structural analogs. Regarding reproducibility, the intra-assay coefficient of variation (CV) was <10%, and the inter-assay CV was <15%. Each experimental group included 10 mice (*n* = 10), and independent biological replicates were derived from individual animals to ensure the statistical robustness of the data. All serum samples were diluted 5-fold prior to detection, in strict accordance with the manufacturer’s instructions. To standardize quantification, standard curves were established using the standards provided with the kits, and the same standard curve was applied across all biological replicates to minimize inter-assay variation. All experimental operations followed the manufacturer’s protocols, and each sample was analyzed in technical triplicates to ensure the reliability of the experimental results.

### 2.7. Histological Analysis

Liver and spleen tissues were collected and immediately fixed in 4% paraformaldehyde, embedded in paraffin, and sectioned at 5 μm. Three distinct sections per organ (*n* = 3 mice per group) were randomly selected for hematoxylin and eosin (H&E) staining. Images were captured using a light microscope (Olympus, Tokyo, Japan). To ensure objectivity, histopathological evaluation was performed in a blinded manner by two independent pathologists. A semi-quantitative scoring system was adopted to assess tissue injury:

Liver Injury Score [33], evaluated based on inflammatory cell infiltration (0 = none, 1 = minimal/scattered, 2 = mild/focal, 3 = moderate/diffuse) and hepatocyte degeneration/disarray (0 = normal, 1 = mild, 2 = moderate, 3 = severe), with a maximum possible score of 6.

Spleen Architecture Score [34], evaluated based on the clarity of the red/white pulp boundary and follicular structure (0 = distinct/normal, 1 = slightly blurred, 2 = moderately blurred/disorganized, 3 = severely indistinct/disrupted).

The final score for each sample represented the mean of the analyzed sections.

### 2.8. Reverse Transcription Quantitative Real-Time PCR (RT-qPCR)

In this study, 50 mg of duodenal tissue was taken and ground in a cryomill (Sevier, Wuhan, China). Total RNA was extracted using TransZol Up Plus RNA Kit (Transgen, Beijing, China), with RNA purity verified by the A260/A280 ratio and RNA integrity assessed using the RNA integrity number (RIN). Samples with an A260/A280 ratio of 1.8–2.1 and acceptable RIN values were defined as qualified samples. Qualified samples were then subjected to genomic DNA removal by the gDNA Removal Reagent (Transgen, Beijing, China). Reverse transcription was then carried out according to the instructions of the TransScript^®^ All-in-One First-Strand cDNA Synthesis SuperMix for qPCR (One-Step gDNA Removal) (Transgen, Beijing, China). The cDNA templates were then synthesized. Real-time fluorescence quantitative PCR was performed using a PerfectStart^®^ Green qPCR SuperMix (Transgen, Beijing, China) on a Gentier 96E/96R real-time PCR system (Tianlong, Xi’an, China). The reaction system consisted of 2×PerfectStart^®^ SYBR Green qPCR Mix (Transgen, Beijing, China). The reaction conditions comprised a pre-denaturation step at 94 °C for 30 s, followed by 5 s at 94 °C and 30 s at 60 °C, with a total of 40 cycles. All primers were synthesized by Beijing Kengke Biotechnology Co. (Beijing, China).

The mRNA expression levels of *Tf*, *Tfrc*, *Fth1*, *Ftl1*, *Hamp*, *Slc11a2*, *Slc40a1*, *Cybrd1*, *HIF-2α*, *TNF-α*, *IL-1β*, *IL-10*, and *IL-6* in duodenal tissue were determined using a Gentier 96E/96R instrument and fluorescent quantitative PCR techniques (Bio-Rad, Hercules, CA, USA). *GAPDH* was used as an internal control, and the expression levels of the different genes were quantified relative to *GAPDH* using the 2^−ΔΔCt^ method. Primer sequences are shown in Supplementary Table S1. To ensure experimental reliability, each biological sample (representing independent animal individuals, *n* = 10) was analyzed with at least three technical replicates (repeated measurements of the same cDNA sample) in RT-qPCR. During data processing, Ct values from technical replicates were averaged for each biological replicate to minimize technical variation; subsequent between-group statistical comparisons were solely based on biological replicates to distinguish and control for differences between biological and technical variations.

### 2.9. 16S rDNA Sequencing

The microbial composition of cecal contents was analyzed by Lianchuan Biological Co., Ltd. (Hangzhou, China). Genomic DNA was extracted from the cecal contents of 50 mice and divided into five groups with 10 mice per group, using the QIAamp DNA Stool Mini Kit (Qiagen, Hilden, Germany), following the manufacturer’s protocol. The integrity and size of the DNA were verified by means of 1% agarose gel electrophoresis, and the concentrations were measured using a NanoDrop 2000 spectrophotometer (Thermo, Wilmington, NC, USA). Sample with an A260/A280 ratio between 1.8 and 2.0 were used for subsequent experiments. The V3-V4 hypervariable region of the 16S rRNA gene was amplified using primers 341F (5′-CCTACGGGNGGCWGCAG-3′) and 806R (5′-GACTACHVGGGTWTCTAATCC-3′). PCR conditions were 98 °C for 30 s; 32 cycles of 98 °C for 10 s, 54 °C for 30 s, 72 °C for 45 s; and a final extension at 72 °C for 10 min. Amplicons (~466 bp) were purified using a 2% agarose gel and the QIAquick PCR Purification Kit (Qiagen, Hilden, Germany). Dual-indexed libraries were constructed using the Illumina Nextera XT Index Kit, ensuring equal sequencing depth across all samples. Sequencing was performed on the Illumina NovaSeq 6000 platform (Illumina, San Diego, CA, USA) with a 2 × 250 bp paired-end configuration. After quality filtering (Q30) and chimera removal, each sample yielded between 48,673 and 96,989 high-quality reads, with an average depth of approximately 78,301 reads per sample. This sequencing depth is sufficient to capture both abundant and rare taxa within the gut microbiota.

Sequences were clustered into Operational Taxonomic Units (OTUs) at a 97% similarity threshold using USEARCH (v11). Taxonomic assignment was conducted using the RDP Classifier (v2.13) against the SILVA database (SSU138). Alpha diversity (e.g., Shannon and Simpson) and beta diversity (e.g., Bray–Curtis) indices were calculated using Mothur (v1.30.2). Differentially abundant taxa were identified using LEfSe (Linear Discriminant Analysis Effect Size). Statistical significance was assessed via the Kruskal–Wallis test (α = 0.05), followed by pairwise Wilcoxon tests. Only taxa with an LDA score > 3.0 were considered significantly different between groups, a threshold that balances statistical significance with biological relevance. No technical replicates were performed for sequencing, but biological replicates were sufficient to ensure statistical power.

### 2.10. Short-Chain Fatty Acid Analysis

Gas chromatography (GC-2010 Plus, Shimadzu, Kyoto, Japan) coupled with a hydrogen (H_2_) flame ionization detector (FID) was employed for quantifying SCFA concentrations in mouse fecal samples. First, 50 mg of fecal samples was thawed on ice, homogenized in 500 μL of ultrapure water, and centrifuged at 12,000 rpm for 10 min at 4 °C. Then, 400 μL of the supernatant was mixed with 40 μL of 2-ethylbutyric acid (internal standard, Sigma-Aldrich, St. Louis, MO, USA) and acidified with 40 μL of 37% HCl. After filtration, the samples were subjected to derivatization prior to GC injection. The chromatographic separation was achieved using an Agilent J&W DB-FFAP capillary column with the following specifications: 0.32 mm inner diameter, 30 m length, and 0.5 μm film thickness (Agilent Technologies, Santa Clara, CA, USA) [35]. The temperature program was as follows: initial temperature at 50 °C (hold 1 min), ramp to 120 °C at 15 °C/min (hold 1 min), ramp to 170 °C at 5 °C/min (hold 3 min), and final ramp to 240 °C at 15 °C/min (hold 5 min). All analyses were performed with *n* = 10 biological replicates and each sample was analyzed in technical triplicates. The SCFA analytes targeted in this assay included acetate, propionate, butyrate, and total SCFAs, with 2-ethylbutyric acid serving as the internal standard for accurate quantification. SCFA concentrations were calculated based on external calibration curves prepared with authentic standard solutions, and the limit of detection (LOD) for each SCFA was determined accordingly; the final SCFA contents were expressed as μmol/g feces and normalized to fecal wet weight.

### 2.11. Statistical Analysis

Data analyses were performed using GraphPad Prism 10.1.2 (GraphPad Software, San Diego, CA, USA). All data were presented as mean ± SEM. Differences between two groups were analyzed using Student’s *t*-test, and comparisons among multiple groups were performed using one-way ANOVA. Prior to ANOVA, the assumptions of normality and homogeneity of variance were verified. For data that did not meet these assumptions, non-parametric tests were applied. *p* < 0.05 was considered statistically significant.

## 3. Results

### 3.1. B. lactis Ca360 Significantly Ameliorates the Symptoms of Iron Deficiency Anemia Induced by a Low-Iron Diet in Mice

**Figure 1:**
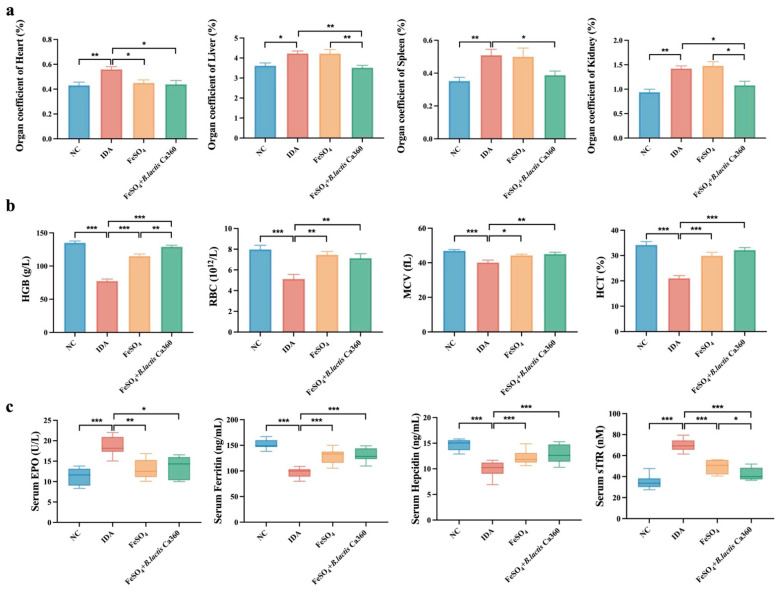
Effect of *B. lactis* Ca360 on symptoms induced by low-iron diet in mice. (**a**) The heart, liver, spleen, and kidney indices were measured. (**b**) HGB, RBC, MCV, and HCT levels were determined by hematological analysis. (**c**) EPO, Ferritin, Hepcidin, and sTfR levels were measured by ELISA. Data are expressed as mean ± SEM (*n* = 10). *, *p* < 0.05; **, *p* < 0.01; ***, *p* < 0.001.

As shown in Figure 1a, organ indices of the heart, liver, and kidneys were significantly elevated in the IDA group compared with the NC group. While FeSO_4_ treatment alone did not significantly reduce these indices, the combination with *B. lactis* Ca360 led to a more pronounced reduction in liver and kidney indices, suggesting a synergistic or additive effect of *B. lactis* Ca360 on alleviating organ enlargement.

Hematological analysis indicated that FeSO_4_ intervention partially restored the reduced levels of HGB, RBC, HCT, and MCV in IDA mice (Figure 1b). HGB is a key index reflecting blood oxygen-carrying capacity and is widely used to evaluate the severity of IDA. In the present study, HGB levels were measured using an automated hematology analyzer, following the manufacturer’s instructions. Both FeSO_4_ treatment alone and the combined interventions significantly increased the aforementioned hematological parameters. Notably, the *B. lactis* Ca360 combined treatment further elevated HGB compared to FeSO_4_ alone and achieved a significantly greater improvement than the *L. plantarum* 299v combined group, highlighting the superior efficacy of *B. lactis* Ca360 in enhancing HGB synthesis.

As shown in Figure 1c, serum levels of EPO and sTfR were significantly increased, while ferritin and hepcidin levels were decreased in the IDA group. Although FeSO_4_ treatment reversed these trends, the addition of *B. lactis* Ca360 resulted in a further significant reduction in sTfR levels beyond that achieved with FeSO_4_ alone. Moreover, the effect of the *B. lactis* Ca360 combination on lowering sTfR levels was significantly stronger than that observed in the *L. plantarum* 299v combined intervention group, indicating that *B. lactis* Ca360 more effectively improves iron availability and utilization.

### 3.2. B. lactis Ca360 Ameliorates IDA-Induced Histopathological Alterations in the Liver and Spleen

**Figure 2:**
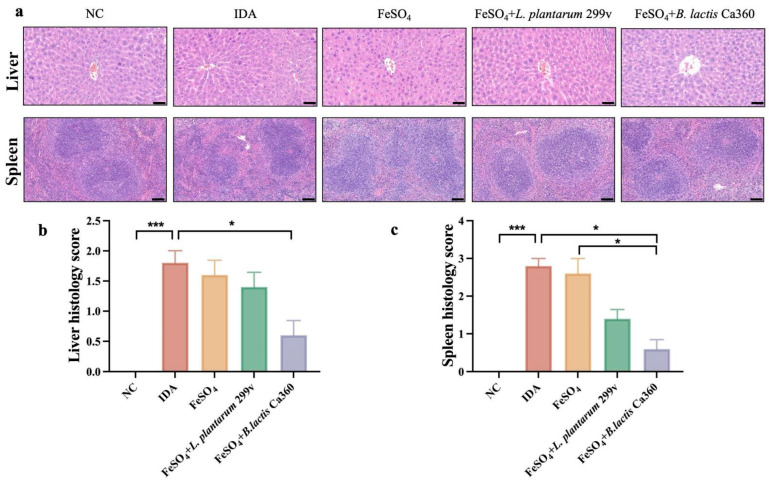
Protective effects of *B. lactis* Ca360 against iron deficiency anemia-induced liver and spleen histopathological alterations. (**a**) Micrographs of H&E-stained sections of liver and spleen tissues (scale bar: 50 μm). (**b**) Liver histology score. (**c**) Spleen histology score. Data are expressed as mean ± SEM (*n* = 3). *, *p* < 0.05; ***, *p* < 0.001.

As shown in Figure 2, liver tissues in the NC group displayed intact architecture with well-organized hepatic cords and no evident inflammatory infiltration. In contrast, mice in the IDA group showed disorganized hepatocyte arrangement, focal cellular swelling, and mild inflammatory infiltration. While FeSO_4_ treatment alone did not markedly improve liver morphology compared to the IDA group, the *B. lactis* Ca360 combined intervention substantially restored hepatic architecture, reduced inflammatory infiltration, and achieved an overall morphology comparable to that of the NC group (Figure 2a). Histopathological scoring further confirmed that the *B. lactis* Ca360 combined intervention significantly decreased liver histology scores in IDA mice (Figure 2b).

Hematoxylin–eosin staining of spleen tissues further revealed clear and well-defined red and white pulp structures in the NC group. In the IDA group, however, the spleen showed loose organization and blurred boundaries between red and white pulp zones. Although FeSO_4_ alone did not produce an obvious improvement, the *B. lactis* Ca360 combined intervention markedly restored the red and white pulp architecture, resulting in a more regular splenic morphology (Figure 2a). Histological scoring of the spleen further supported these observations (Figure 2c). Notably, while the *L. plantarum* 299v combination also promoted splenic recovery, its effect was less pronounced than that of the *B. lactis* Ca360 combined intervention.

### 3.3. B. lactis Ca360-Mediated Modulation of Duodenal Iron Metabolism and Inflammation in IDA Mice

**Figure 3:**
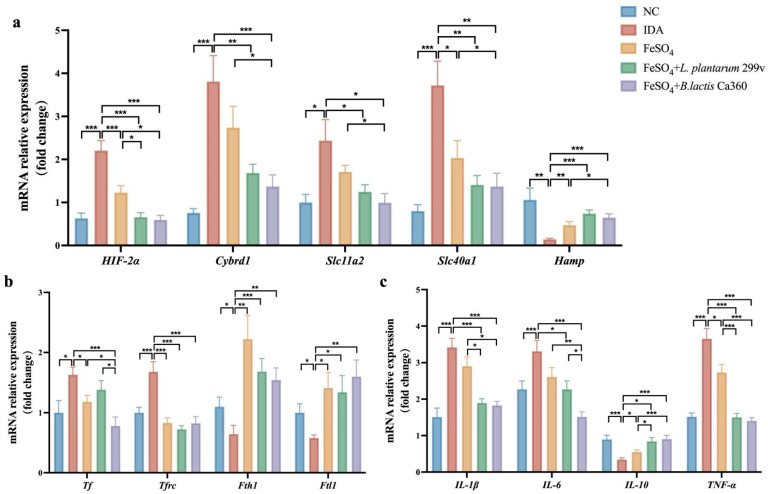
*B. lactis* Ca360 modulates the iron metabolism and inflammatory responses in the duodenal tissues. Relative mRNA expression levels of (**a**) *HIF-2α*, *Cybrd1*, *Slc11a2*, *Slc40a1*, and *Hamp*; (**b**) *Tf*, *Tfrc*, *Fth1*, and *Ftl1*; and (**c**) *IL-1β*, *IL-6*, *IL-10*, and *TNF-α*. Data are expressed as mean ± SEM (*n* = 10). *, *p* < 0.05; **, *p* < 0.01; ***, *p* < 0.001.

Figure 3a,b present changes in the expression of genes involved in iron absorption, transport, and storage within the duodenum of IDA mice. Compared to the NC group, duodenum expression of *HIF-2α*, *Cybrd1*, *Slc11a2*, and *Slc40a1* was significantly upregulated in IDA mice, while *Hamp* expression was markedly downregulated. *Hamp* is the master regulator of systemic iron homeostasis, and its downregulation in IDA mice represents a compensatory physiological response to insufficient body iron stores, aiming to enhance intestinal iron absorption and mobilize iron from tissues to maintain systemic iron availability. After FeSO_4_ monotherapy, *HIF-2α* and *Slc40a1* expression in the duodenum was significantly downregulated, whereas *Hamp* expression was substantially upregulated. The recovery of *Hamp* expression reflects the restoration of iron homeostasis, as increased *Hamp* inhibits excessive iron absorption and prevents iron overload upon iron repletion. Nevertheless, the expression levels of these genes were still distinct from those observed in the NC group. In the *B. lactis* Ca360 combined intervention group, the expression levels of *HIF-2α*, *Cybrd1*, *Slc11a2*, and *Slc40a1* were significantly downregulated compared with those in the IDA group, with its effect slightly surpassing that of the *L. plantarum* 299v combination treatment. Notably, the *B. lactis* Ca360 combined intervention also effectively upregulated *Hamp* expression, further indicating its ability to normalize the central regulatory axis of iron metabolism. Regarding genes associated with iron transport and storage, duodenal *Tf* and *Tfrc* expression was significantly upregulated in the IDA group compared with the NC group, while *Fth1* and *Ftl1* expression was significantly downregulated. Both FeSO_4_ monotherapy and *B. lactis* Ca360 combination treatment significantly improved the expression profiles of these genes, with *B. lactis* Ca360 combination treatment yielding the most favorable outcome.

Furthermore, analysis of inflammatory genes revealed that, compared to the NC group, the IDA group exhibited significantly upregulated expression of *IL-1β*, *IL-6*, and *TNF-α*, accompanied by a significant decrease *IL-10* expression. Following combination treatments, the expression of these pro-inflammatory genes was significantly downregulated, while *IL-10* expression was significantly upregulated. Importantly, the *B. lactis* Ca360 combination treatment demonstrated significantly greater efficacy than FeSO_4_ monotherapy in regulating *IL-1β* and *IL-10* expression and outperformed the *L. plantarum* 299v combination treatment in modulating *IL-6* expression (Figure 3c).

### 3.4. B. lactis Ca360 Reshapes Gut Microbiota Composition and Enhances SCFA Production in IDA Mice

**Figure 4:**
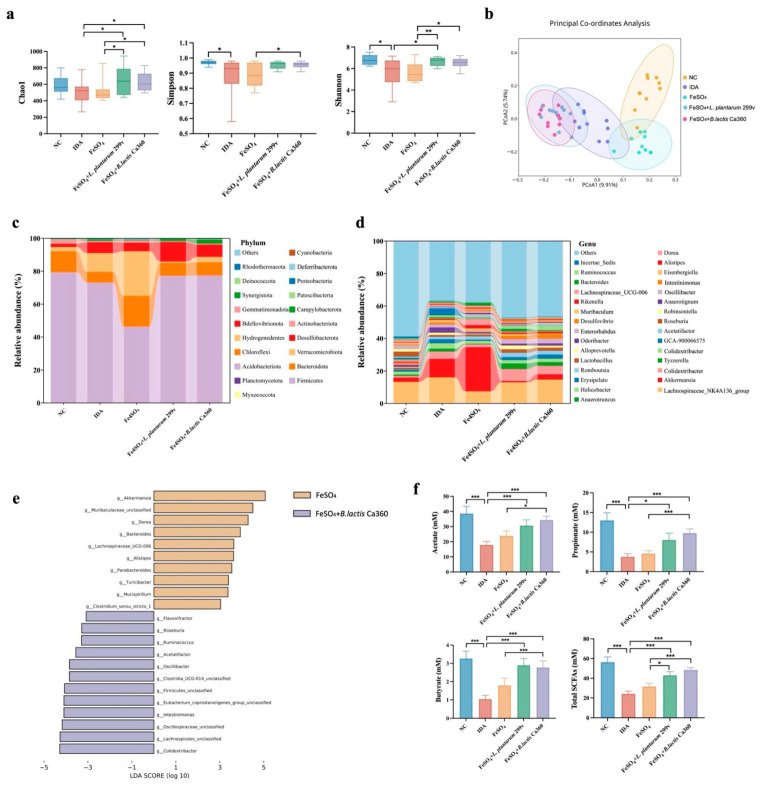
Functional changes in the gut microbiota of *B. lactis* Ca360-treated mice. (**a**) The α-diversity analysis of Chao1, Shannon, and Simpson indices. (**b**) The β-diversity analysis. (**c**) Microbial community bar plot by phylum. (**d**) Microbial community composition at the genus level. (**e**) Bacterial taxa that were significantly different between the two groups were identified by LEfSe, LDA score ≥ 3.0. (**f**) Analysis of fecal SCFAs including acetate, propionate, butyrate, and total SCFAs. Data are expressed as mean ± SEM (*n* = 10). *, *p* < 0.05; **, *p* < 0.01; ***, *p* < 0.001.

The gut microbiota composition of cecal contents was analyzed by 16S rDNA sequencing, with the results presented in Figure 4. The α-diversity analysis showed a significant reduction in the Shannon and Simpson diversity indices in the IDA group compared to the NC group. In contrast, the *B. lactis* Ca360 combination treatment significantly elevated the Chao1 and Simpson indices relative to the IDA group and further increased the Chao1 and Shannon indices compared to the FeSO_4_ alone group (Figure 4a). The β-diversity analysis using the Jaccard distance matrix and principal coordinates analysis (PCoA) revealed clear separation of both the IDA group and the *B. lactis* Ca360 combination treatment group from the NC group along the PCoA1 axis (Figure 4b). Further taxonomic analysis at the phylum and genus levels indicated a marked disruption of gut microbial structure in IDA mice. Specifically, the relative abundances of Bacteroidota and Actinobacteriota were significantly lower than those in the NC group. The *B. lactis* Ca360 combination treatment notably increased the relative abundances of Bacteroidota and Campylobacterota (Figure 4c). At the genus level, this intervention decreased the relative abundances of *Akkermansia, Clostridium*, *Anaerotignum*, *Erysipelatoclostridium*, and *Eisenbergiella* in IDA mice, while elevating the abundances of *Colidextribacter*, *Oscillibacter*, *Intestinimonas*, and *Helicobacter* (Figure 4d). LEfSe analysis further indicated that the *B. lactis* Ca360 combination treatment was significantly enriched in *Colidextribacter*, *Lachnospirales_unclassified*, *Oscillospiraceae_unclassified*, *Intestinimonas*, *Eubacterium_coprostanoligenes_group_unclassified*, *Firmicutes_unclassified*, *Clostridia_UCG-014_unclassified*, *Oscillibacter*, *Acetatifactor*, *Ruminococcus*, Roseburia, and *Flavonifractor* compared to the FeSO_4_ group (Figure 4e). Analysis of SCFAs revealed that, compared with the IDA group, the *B. lactis* Ca360 combination treatment significantly increased the levels of acetate, propionate, butyrate, and total SCFAs. Moreover, these levels were also significantly higher than those observed in the FeSO_4_ alone group (Figure 4f).

### 3.5. Spearman Correlation Analysis

**Figure 5:**
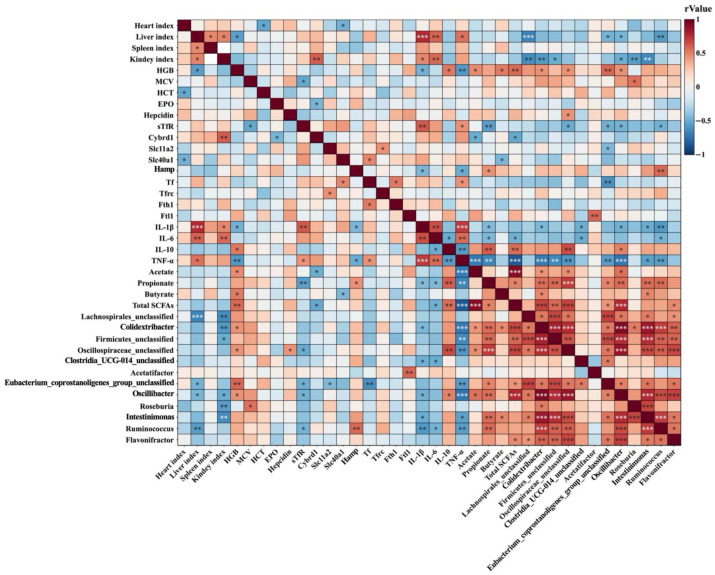
Spearman correlation analysis. Heatmap analysis of correlations among hematological parameters, iron metabolism indicators, inflammatory markers, SCFA levels, and gut-enriched probiotic bacteria. Red and blue represent positive and negative correlations, respectively. The darker the color, the stronger the correlation. *p*-values were adjusted using the Benjamini–Hochberg procedure to control the false discovery rate (FDR). An adjusted *p*-value < 0.05 was considered statistically significant. *N* = 10, *, *p* < 0.05; **, *p* < 0.01; ***, *p* < 0.001.

The results of a Spearman correlation analysis are shown in Figure 5. Bacterial genera significantly enriched in the combined intervention group were correlated with key host variables, including iron metabolism markers, hematological parameters, inflammatory markers, and SCFA levels. Specifically, *Lachnospirales_unclassified*, *Colidextribacter*, *Acetatifactor*, *Firmicutes_unclassified*, *Oscillospiraceae_unclassified*, *Clostridia_UCG-014_unclassified*, *Eubacterium_coprostanoligenes_group_unclassified*, *Ruminococcus*, *Roseburia*, *Oscillibacter*, *Intestinimonas*, and *Flavonifractor* showed significant positive correlations with at least one of HGB, MCV, hepcidin, *Hamp*, *Ftl1*, *IL-10*, acetate, propionate, butyrate, and total SCFAs, while being negatively correlated with at least one of the following indicators: organ indices (liver and kidney), sTfR, *Slc11a2*, *Tf*, *IL-1β*, *IL-6*, and *TNF-α*.

## 4. Discussion

Iron deficiency anemia (IDA) develops due to disrupted systemic iron homeostasis, driven collectively by inadequate iron intake [36], impaired intestinal absorption [37], and inflammation-associated iron restriction [38]. Growing evidence indicates that gut microbiota play key roles in host nutrient metabolism, hematopoietic function, and the modulation of inflammatory responses and iron-regulatory pathways [39]. Nevertheless, the systemic mechanisms by which the gut microbiota contribute to probiotic-assisted iron supplementation remain poorly defined. Among probiotics studied for IDA intervention, *L. plantarum* 299v has been reported to modestly improve iron bioavailability by regulating intestinal epithelial integrity, but its efficacy in restoring systemic iron homeostasis and alleviating IDA-associated organ damage is limited [40]. In the present study, we focused on *B. lactis* Ca360 as the investigational strain and systematically delineated its coordinated regulatory effects on the gut microbiota, inflammatory responses, and iron metabolism networks under iron-deficient conditions. Our findings demonstrated that *B. lactis* Ca360 effectively restores iron homeostasis, reshapes gut microbial composition, and mitigates inflammatory markers in IDA mice, providing experimental and mechanistic support for probiotic assisted iron supplementation in the management of iron deficiency anemia.

Analysis of organ indices and hematological parameters showed that an iron-deficient diet markedly increased the indices of heart, liver, and kidneys in mice, along with marked reductions in HGB, RBC, HCT, and MCV, demonstrating typical characteristics of iron deficiency anemia [41]. These findings suggest that iron deficiency not only compromises hematopoietic function but also leads to compensatory organ enlargement and systemic metabolic stress [42], which is highly consistent with earlier animal and clinical studies reporting increased organ mass [43], impaired oxygen transport, and disturbed energy metabolism under iron-deficient conditions [44]. Following FeSO_4_ intervention, most of these abnormal parameters were significantly improved, confirming the effectiveness of iron supplementation in reversing anemia and alleviating organ burden. Notably, the combination of FeSO_4_ and *B. lactis* Ca360 demonstrated superior efficacy in lowering liver and kidney indices and improving HGB and RBC levels. Compared with the *L. plantarum* 299v combination group, the *B. lactis* Ca360 combined iron supplement group showed improvements in lowering the spleen index, increasing HGB levels, and decreasing sTfR levels that were closer to the NC group, consistent with previous reports indicating that probiotics can enhance the therapeutic efficacy of iron supplementation [45]. Furthermore, iron metabolism-related parameters revealed that in the IDA group, EPO and sTfR levels were significantly elevated, whereas ferritin and hepcidin levels were markedly decreased. This is consistent with the classical regulatory response to iron deficiency, in which hepcidin expression is suppressed to enhance iron mobilization and erythropoietic stress is increased to maintain iron availability [46]. After intervention, EPO and sTfR levels decreased, whereas ferritin and hepcidin levels increased, indicating a gradual restoration of iron homeostasis. Importantly, the combined intervention was significantly more effective than FeSO_4_ alone in lowering sTfR levels, further supporting a synergistic role of *B. lactis* Ca360 in improving iron utilization, alleviating functional iron deficiency, and promoting recovery from anemia.

Histologically, the liver and spleen are central organs for iron homeostasis, serving as the primary sites for iron recycling, storage, and erythrophagocytosis [47]. Therefore, assessing the histological integrity of these organs provides crucial insights into the systemic pathological consequences of IDA and the efficacy of therapeutic interventions. In the present study, an iron-deficient diet induced pronounced structural damage in both organs, indicating that iron deficiency not only impaired systemic hematopoietic function but also disrupted tissue-level organ homeostasis, as evidenced by pronounced hepatic lesions. In IDA mice, liver sections displayed disorganized hepatocyte arrangement, nuclear atrophy, and mild inflammatory infiltration, suggesting that iron deficiency may compromise liver integrity through metabolic stress and low-grade inflammation, aligning with recent findings on oxidative stress-mediated hepatic injury [48]. Notably, while FeSO_4_ alone exerted limited efficacy in restoring hepatic architecture, the combination with *B. lactis* Ca360 markedly improved liver integrity and reduced inflammatory infiltration, demonstrating *B. lactis* Ca360’s active role in promoting hepatic recovery. Similarly, the spleen—a vital hematopoietic and immune organ—exhibited blurred red and white pulp boundaries under iron-deficient conditions, reflecting compensatory responses to anemia [49]. While FeSO_4_ treatment alone was insufficient in reversing these structural alterations, the combined intervention with *B. lactis* Ca360 successfully restored splenic architecture, aligning with previous findings on the synergistic effect of combined iron supplementation [50]. Collectively, these observations indicate that iron supplementation alone is insufficient to fully reverse IDA-associated organ damage. In contrast, the combined administration of *B. lactis* Ca360 not only potentiates the therapeutic efficacy of iron supplementation but also promotes tissue structural repair in both the liver and spleen. This synergistic effect likely underpins the observed restoration of systemic metabolic and immune homeostasis.

The regulation of iron absorption, transport, and storage is essential for maintaining systemic iron homeostasis, with the duodenum playing a central role in dietary iron utilization through transcriptional regulation of key iron metabolism-related genes [51]. In the present study, iron deficiency markedly upregulated duodenal *HIF-2α* and its downstream targets *Cybrd1*, *Slc11a2*, and *Slc40a1*, while *Hamp* expression was significantly downregulated, suggesting a compensatory activation of the *HIF-2α*-dependent intestinal iron absorption pathway to enhance iron uptake. *Cybrd1* is localized on the brush-border membrane of intestinal enterocytes and functions as a membrane-bound ferric reductase that reduces luminal non-heme iron from Fe^3+^ to Fe^2+^. This reduction step provides the essential substrate for *Slc11a2*-mediated uptake of ferrous iron, thereby forming a critical entry step for intestinal non-heme iron absorption in the duodenum. Under iron-deficient conditions, *HIF-2α* upregulates the expression of *Cybrd1* and *Slc11a2*, thereby enhancing ferric iron reduction and subsequent transmembrane transport of Fe^2+^. In coordination with *Slc40a1*, this regulatory axis facilitates the export of absorbed iron into the circulation, constituting an adaptive mechanism that increases intestinal iron absorption. This regulatory pattern is consistent with previous studies demonstrating that *HIF-2α* acts as a key transcriptional regulator of intestinal iron absorption [52]. FeSO_4_ treatment alone partially reversed these gene expression abnormalities; however, the expression levels failed to return to levels observed in the NC group, indicating limited efficacy of iron supplementation alone in fully restoring intestinal iron metabolic homeostasis [53]. In contrast, the combined intervention significantly normalized these expression profiles, suggesting that *B. lactis* Ca360 actively promotes coordinated regulation of intestinal iron absorption. Consistently, expression of *Tf* and *Tfrc* was significantly elevated in IDA mice, while *Fth1* and *Ftl1* levels were markedly reduced, reflecting increased cellular iron demand and restricted iron storage, molecular features consistent with functional iron deficiency [54]. Both intervention regimens significantly ameliorated these alterations. Furthermore, iron deficiency was accompanied by elevated hepatic expression of pro-inflammatory genes (*IL-1β*, *IL-6*, and *TNF-α*) and decreased *IL-10* expression, indicative of a systemic low-grade inflammation state [55]. The combined intervention with *B. lactis* Ca360 not only significantly suppressed pro-inflammatory gene expression but also restored anti-inflammatory signaling, outperforming both FeSO_4_ treatment alone and *L. plantarum* 299v combination treatment. Notably, *Tf* and *IL-6* levels were significantly lower in the *B. lactis* Ca360 combination group than in the *L. plantarum* 299v combination group. These findings support a potential mechanism whereby *B. lactis* Ca360 contributes to iron homeostasis indirectly by modulating the inflammatory microenvironment, aligning with previous reports that probiotics can influence iron metabolism through the regulation of inflammatory responses [56], while highlighting *B. lactis* Ca360’s more potent anti-inflammatory capacity compared with *L. plantarum* 299v.

Previous studies have demonstrated that iron deficiency can trigger gut microbiota dysbiosis, which may further reduce iron absorption efficiency, thereby establishing a vicious cycle [57]. Although *L. plantarum* 299v has been shown to slightly increase gut microbial diversity and elevate SCFA concentrations in healthy individuals [58], direct evidence for its profound restructuring of gut microbial composition, such as the significant enrichment of SCFA-producing bacteria, remains lacking in IDA models. The results of this study suggest that *B. lactis* Ca360 does not act merely as an iron supplement, but rather modulates gut microbiota composition to coordinately improve the intestinal microenvironment linked to iron metabolism. Following the combined intervention, gut microbial diversity and community structure displayed a clear trend toward recovery to a normal state, suggesting that *B. lactis* Ca360 helps restore the disrupted intestinal ecological balance under iron-deficient conditions [59]. Accordingly, the observed restoration of microbiota structure likely establishes an essential ecological foundation for the recovery of systemic iron homeostasis.

At the functional level, the core genera enriched following the combined intervention, primarily *Intestinimonas*, *Oscillibacter*, *Roseburia*, *Ruminococcus*, *Odoribacter*, and *Acetatifactor*, are predominantly characterized by strict anaerobic metabolism and recognized as major producers of SCFAs, particularly butyrate and acetate [60]. Consistent with the existing literature, core enriched genera such as *Roseburia* [61], *Ruminococcus* [62], and *Intestinimonas* [63] are established butyrate-producing bacteria that ferment dietary fiber and polysaccharides under anaerobic conditions, providing a primary energy source for colonic epithelial cells. Meanwhile, *Oscillibacter* [64] and *Odoribacter* [65] are primarily linked to acetate and propionate production and participate in anti-inflammatory metabolic pathways. Emerging evidence suggests that microbiota-derived metabolites, particularly SCFAs, can influence intestinal hypoxia signaling and iron metabolism [66]. *HIF-2α* is a key oxygen- and iron-sensitive transcription factor that regulates genes involved in intestinal iron absorption, including *Cybrd1*, *Slc11a2*, and *Slc40a1* [67]. SCFAs produced by anaerobic commensal bacteria may modulate *HIF-2α* stability and transcriptional activity through several mechanisms, including activation of G-protein-coupled receptors (*GPR41/43*), inhibition of histone deacetylases, and regulation of cellular energy metabolism and redox balance [68]. Consistent with these mechanisms, enrichment of SCFA-producing taxa following *B. lactis* Ca360 intervention may attenuate excessive compensatory activation of *HIF-2α* under iron-deficient conditions. This modulation likely contributes to normalization of intestinal iron absorption signaling and restoration of systemic iron homeostasis.

Notably, *L. plantarum* 299v fails to enrich these key SCFA-producing genera, whereas the selective enrichment of these SCFA-producers helps enhance the expression of intestinal tight-junction proteins [69], reduce intestinal permeability, and strengthen barrier integrity [70], collectively creating a more stable microenvironment conducive to transepithelial iron transport [71]. Corresponding to these functional shifts, quantitative SCFA analysis revealed significantly elevated levels of acetate, propionate, and butyrate in the *B. lactis* Ca360 co-intervention group compared to the FeSO_4_ monotherapy group. No significant differences were observed in the *L. plantarum* 299v co-intervention group. This outcome highlights the unique advantage of *B. lactis* Ca360 in promoting SCFA production through microbiota remodeling.

Finally, an integrative Spearman correlation analysis was conducted to systematically assess the relationships among all measured variables. These results revealed, at a systemic level, intrinsic connections between the gut microbiota changes induced by the combined intervention and concurrent improvements in iron metabolism, inflammatory markers, and anemia-related phenotypes. These associations are weaker or absent in *L. plantarum* 299v combination treatment. Key strictly anaerobic genera that were significantly enriched after the combined intervention, such as *Ruminococcus*, *Roseburia*, *Acetatifactor*, *Intestinimonas*, *Eubacterium_coprostanoligenes_group_unclassified*, and *Oscillibacter*, are established producers of SCFAs. The abundances of these taxa and their associated SCFA metabolites were consistently positively correlated with hemoglobin levels and erythropoiesis-related parameters, suggesting that an SCFA-centered metabolic environment critically supports hematopoietic recovery. Concurrently, these SCFA-producing bacteria were negatively correlated with hepatic pro-inflammatory gene expression and positively correlated with anti-inflammatory markers, indicating that gut microbiota remodeling may contribute to the attenuation of hepatic inflammation. This anti-inflammatory regulatory network proved more potent than that mediated by *L. plantarum* 299v. This anti-inflammatory effect likely represents a key potential mechanism through which the combined intervention suppresses systemic inflammation responses and modulates iron homeostasis. Specifically, at the level of iron metabolism regulation, SCFA-related genera such as *Ruminococcus* and *Roseburia* exhibited significant negative correlations with serum EPO, sTfR, as well as the iron demand-related genes *Tfrc* and *Tf*. These associations suggest that reshaping the gut microbiota composition may help suppress the excessive host iron demand signaling characteristic of iron deficiency. Furthermore, these genera were also significantly negatively correlated with duodenal *HIF-2α* and its downstream iron absorption genes (*Cybrd1*, *Slc11a2*, and *Slc40a1*), indicating a concomitant reduction in compensatory intestinal iron absorption activation. In contrast, significant positive correlations between these taxa and serum hepcidin as well as *Hamp* expression suggested a restoration of iron regulatory feedback. Collectively, these associations indicate that gut microbiota remodeling promotes recovery of systemic iron metabolism through coordinated regulation of multiple iron-related regulatory nodes. Furthermore, the dominant bacterial genera were significantly negatively correlated with indices of the heart, liver, and spleen, linking gut microbiota improvement to a reduction in systemic metabolic burden and alleviation of organ compensatory stress.

In summary, correlation network analysis provides integrative evidence that *B. lactis* Ca360 facilitates the improvement of IDA phenotypes by reshaping gut microbiota that are enriched in SCFA-producing and anti-inflammatory taxa, thereby coordinately regulating intestinal iron absorption, hepatic inflammatory status, and systemic iron homeostasis.

## 5. Conclusions

This study investigated the therapeutic potential of *B. lactis* Ca360 in IDA. We found that *B. lactis* Ca360 synergistically enhanced the efficacy of iron supplementation in IDA mice, likely by reshaping the gut microbiota toward anaerobic communities that produce SCFAs. This remodeling restored intestinal iron metabolism, alleviated hepatic inflammation, and improved systemic iron homeostasis, which may represent a key mechanism underlying the amelioration of IDA. These findings provide novel mechanistic insights into the synergistic regulatory effects of *B. lactis* Ca360 combined with iron therapy, highlighting the pivotal roles of gut microbiota reprogramming and SCFAs, and offering a new perspective for the use of adjuvants for iron therapy. Despite these contributions, this study has certain limitations: the results were obtained in a mouse model and require validation in humans; the mechanistic analysis was mainly based on gene expression and correlation, and further protein-level and causal studies are needed. These insights provide novel experimental evidence and mechanistic support for the use of probiotics as an effective adjunct strategy in iron supplementation regimens.

## 6. Patents

The work reported in this study has been granted an invention patent, with specific details as follows: title of the invention patent: “A Strain of *Lactobacillus Plantarum* Promoting Mineral Absorption and Transport and Its Application”; patentee: Inner Mongolia Mengniu Dairy (Group) Co., Ltd.; patent No.: ZL202510791324.X; certificate No.: No. 8280274; announcement date of grant: 19 September 2025.

## Data Availability

The original contributions presented in the study are included in the article/Supplementary Materials, further inquiries can be directed to the corresponding authors.

## Supplementary Materials

The following supporting information can be downloaded at https://www.mdpi.com/article/10.3390/nu18060900/s1, Table S1: Primer sequences used in RT-qPCR assays.

## Abbreviations

The following abbreviations are used in this manuscript:

IDA	Iron deficiency anemia
*B. lactis* Ca360	*Bifidobacterium animalis* subsp. *lactis* Ca360
*L. plantarum* 299v	*Lactobacillus plantarum* 299v
SCFA	Short-chain fatty acid
FeSO_4_	Ferrous sulfate
PBS	Phosphate-buffered saline
HGB	Hemoglobin content
RBC	Red blood cells
HCT	Hematocrit
MCV	Mean corpuscular volume
ELISA	Enzyme-linked immunosorbent assay
EPO	Erythropoietin
sTfR	Soluble transferrin receptor
H&E	Hematoxylin and Eosin
RT-qPCR	Real-time quantitative polymerase chain reaction
*DcytB*	Duodenal cytochrome B
*DMT1*	Divalent metal transporter 1
H_2_	Hydrogen
PCoA	Principal coordinate analysis
