# Supplementary material for: Bifidobacterium animalis subsp. lactis Ca360 Promotes Oral Iron Repletion, Alters the Gut Microbiota, and Regulates Host Metabolism and Inflammatory Status in a Murine Model of Iron Deficiency Anemia Caused by a Low-Iron Diet
Source: Nutrients. 2026 Mar 12;18(6):900. doi: 10.3390/nu18060900 (PMC13029082; doi:10.3390/nu18060900)
Supplement: Supplementary file 1 [file nutrients-18-00900-s001.zip › nutrients-4128794-supplementary.pdf]

Table S1. Primer sequences used in RT-qPCR assays.

Gene	Organism	Sequence (5'-3')	Tm℃
HIF-2α	Mouse	F: CTGAGGAAGGAGAAATCCCGT	57.42
		R: TGTGTCCGAAGGAAGCTGATG	58.68
Cybrd1	Mouse	F: CAGTGATTGCGACGGTTCTCA	62.6
		R: ATGAATGGTACGAGGGGTGTT	60.9
Slc11a2	Mouse	F: TGTTTGATTGCATTGGGTCTG	54.76
		R: CGCTCAGCAGGACTTTCGAG	61.51
Slc40a1	Mouse	F: CTTGCTCTGGAAGGTTTACC	54.15
		R: TGGAGTCTTTCTCACCCATT	53.41
Hamp	Mouse	F: CCTGAGCAGCACCACCTATC	58.38
		R: TGCAACAGATACCACACTGGG	57
Tf	Mouse	F: GCTGTCCCTGACAAAACGGT	56.81
		R: CGGAAGGACGGTCTTCATGTG	58.79
Tfrc	Mouse	F: GTGGAGTATCACTTCCTGTCGC	58.37
		R: CCCCAGAAGATATGTCGGAAAGG	58.82
Fth1	Mouse	F: CAAGTGCGCCAGAACTACCA	58.55
		R: GCCACATCATCTCGGTCAAAA	56.36
Ftl1	Mouse	F: CCATCTGACCAACCTCCGC	57.32
		R: CGCTCAAAGAGATACTCGCC	58.75
IL-1β	Mouse	F: GCAACTGTTCCTGAACTCAACT	60.7
		R: ATCTTTTGGGGTCCGTCAACT	61.4
IL-6	Mouse	F: GGGACTGATGCTGGTGACAA	59.96
		R: ACAGGTCTGTTGGGAGTGGT	60.69
IL-10	Mouse	F: GCTCTTACTGACTGGCATGAG	60.2
		R: CGCAGCTCTAGGAGCATGTG	62.7
TNF-α	Mouse	F: CCCTCACACTCAGATCATCTTCT	60.9
		R: GCTACGACGTGGGCTACAG	62.1
Gapdh	Mouse	F: TGACCTCAACTACATGGTCT	55.48
		R: CTTCCCATTCTCGGCCTTG	58.21
